# Tackling illicit tobacco during COVID-19 pandemic

**DOI:** 10.18332/tid/137086

**Published:** 2021-06-10

**Authors:** Hana Ross, Luk Joossens

**Affiliations:** 1 Research Unit on the Economics of Excisable Products, University of Cape Town, Cape Town, South Africa; 2 Illicit Tobacco Trade Expert, Leuven, Belgium

**Keywords:** COVID-19, illicit trade, tobacco

*The 18th WCTOH has announced its plan to hold a virtual ‘Leadership Summit on Tobacco Control’ in 2021. Ahead of the Summit it is planned to hold a series of open-access webinars to provide on-going access to expert opinion on issues of importance to the Tobacco Control community. The last webinar of the series ‘Tackling illicit tobacco during COVID-19 pandemic’ was held 13 April 2021.*

*The webinar aimed at raising awareness about the challenges faced by governments to address the problem of illicit trade, and the importance of adopting and implementing the Protocol in addressing those challenges, especially during the fight with the pandemic that is global in nature.*

The Protocol to Eliminate Illicit trade in Tobacco Products, also known as the Illicit Tobacco Trade Protocol (ITP), is the first protocol to the WHO Framework Convention on Tobacco Control (WHO FCTC), and a new international treaty in its own right. It was adopted by consensus on 12 November 2012 at the fifth session of the Conference of the Parties (COP) to the WHO FCTC (Seoul, Republic of Korea, 12–17 November 2012). The Protocol builds upon and complements Article 15 of the WHO FCTC, which addresses means of countering illicit trade in tobacco products, a key aspect of a comprehensive tobacco control policy.

The ITP is an international treaty with the objective of eliminating all forms of illicit trade in tobacco products through a package of measures to be taken by countries acting in cooperation with each other: it is a global solution to a global problem^1^.

By March 2021, there were 62 parties to the Protocol. A current list of signatories and policies can be found in the UN Treaty Collection entry^2^.

The list of parties is impressive and includes countries such as Brazil, Nigeria, Kenya, Egypt, Turkey, Islamic Republic of Iran, Saudi Arabia, India, Pakistan, UK, European Union, Germany, and France. However, some important countries are still missing such as COP host countries South Africa, Russian Federation, Republic of Korea, and China which was among the first signatories on 10 January 2013.

The first Meeting of the Parties (MOP1) to the Protocol took place 8–10 October 2018. The second MOP should have taken place in November 2020, but was postponed due to COVID-19.

The opportunities presented by this new international treaty are evident. The elimination of the global illicit tobacco trade would lead to more revenue and less tobacco sales. Using 2007 data, the total lost revenue to illicit cigarette trade was estimated at about US$40.5 billion a year. If this illicit trade were eliminated governments would gain at least $31.3 billion a year, and expect a fall in consumption of 2%^3^. New research, released in 2020, confirmed that the elimination of illicit cigarettes in 36 countries with solid data from independent sources on illicit trade would reduce total cigarette consumption by 1.9%. The study confirmed also that tax revenues from the legal sale of cigarettes would increase by 11.2% across those countries^4^. Elimination of illicit tobacco trade can have a positive impact on public finances and should be part of the COVID-19 recovery plan in each party.

But the challenges cannot be ignored either. Less economic growth, more financial constraints, travel restrictions and the subsequent annulment of all physical meetings as a result of COVID-19 made the implementation of the Protocol more difficult.

While some countries, such as India, are trying to move forward with the implementation of the Protocol, others have noted increased levels of illicit trade during the pandemic.

## India

The implementation of the Protocol became a pressing issue for the government of India where the tobacco industry opposes tobacco tax increases by pointing to the rising level of illicit trade. Even though the industry claims that about 24% of the tobacco market consist of illegal products, reputable studies point to 3–6% illicit market penetration.

Since its accession to the ITP in June 2018, India began to work towards the establishment of a tracking and tracing (T&T) regime and placing unique markings on each cigarette pack. With the 2023 deadline approaching, India set up a working group to chart out the modalities of the T&T that India should adopt. In the first phase, the working group studied the T&T system in various countries, but then focused more narrowly on Kenya, Brazil and the European Union. This led to the drafting of a detailed legal proposal for the T&T administration and infrastructure followed by the stakeholders’ engagement. The third step is going to involve issuing of new legal provisions and setting up the appropriate administration. This will be followed by issuing the tender, awarding the contract, test piloting the system and finally the full launch.

Fortunately, India can draw on its prior experience with the T&T for selected pharmaceuticals and liquor. In many countries, it is more efficient and cheaper to implement a T&T system for multiple products. The cigarette T&T will be managed by the Central Excise office with a possibility to involve officers from other departments/ministries to aid the enforcement.

## South Africa

One country that experienced an increase in illicit trade during the pandemic is South Africa, where the government banned the sale of all tobacco and vaping products for almost 5 months in 2020 as part of a national COVID-19 lockdown. The rationale was to prevent more serious illnesses among smokers infected by COVID-19, thereby easing the pressure on the healthcare sector overwhelmed by COVID-19 cases. The Minister of Health subsequently added another justification in response to criticism: to prevent smokers from sharing a cigarette, a common practice among poor smokers in South Africa.

The only other countries with a sales ban during lockdowns were Botswana and India, where these bans lasted 12 weeks and 6 weeks, respectively.

Had the Minister consulted with the Research Unit on the Economics Excisable Products (REEP)^5^, a premier research group focusing on tobacco control and illicit trade economics, and based in her own country, she would have been advised against the ban since there was no strategy in place to combat the illicit cigarette trade, such as a T&T system and a control mechanism of export practices of the cigarette manufacturers. The Unit’s studies over the last 5 years clearly demonstrate the vulnerability of the South African cigarette market to tax evasion, given that the lucrative market for illegal cigarettes prior to the lockdown occupied 30–40% of the total cigarette market^6^.

A REEP study^7^ found that even though about 9% of pre-lockdown smokers quit, and the majority intended to stay quit (about 6% of pre-lockdown smokers), the overwhelming majority of continuing smokers (93%) were able to purchase cigarettes despite the sales ban. Unlike illicit cigarette users prior to the lockdown, these smokers paid a hefty premium for their illegal products: the average price of cigarettes went up by 250% compared to pre-lockdown prices. Higher prices motivated 46% of smokers to switch from expensive international brands to the relatively cheaper local/ smuggled brands. Poor smokers reported more not less cigarette sharing, and all smokers were subject to a higher risk of COVID-19 exposure as they were searching for cigarettes during the sales ban.

The illicit cigarette market during the ban became so profitable that it attracted an array of new actors, including crime syndicates and ordinary civilians seeking to make a living amid a worsening economic crisis exacerbated by the pandemic. The local cigarette manufacturers that were allowed to produce for export during the ban increased the volume of cigarettes sent abroad, with some evidence that these products never left the country or were smuggled back in order to generate a huge profit for the manufacturers. When the sales ban eventually ended, the government was short of South African Rand 5.8 billion^8^ (about US$ 423 million) in tobacco tax revenue. It also lost a court case, brought by the tobacco industry, regarding the constitutionality of the ban.

The new illicit distribution networks established during the ban will be hard to control, especially thanks to the extraordinary profits made that can be now invested in circumventing tax administration^9^.

## The ITP: time is running out

The World Bank recently released a report on ‘Confronting Illicit Tobacco Trade: A Global Review of Country Experiences’. The report demonstrates that reducing illicit trade in tobacco products is both possible and critical, whether viewed from the perspective of public health, public finance, governance, or equity^10^. The ITP contains strong measures to control supply chain. But time is running out. The tracking and tracing obligations under the ITP for instance will come into force in September 2023, but many parties still do not have such a system in place, and it takes several years to implement it. A global system is more than the sum of existing domestic systems which do not use the same standards to encode, register or monitor events when the products move along the supply chain globally. The exchange of data globally between parties will only be possible if a decision is taken to establish the global information-sharing focal point at the next MOP in November 2021.

An important challenge is the large difference in technical capacity of customs and enforcement authorities in different regions of the world. Half of the ITP parties are small or low- or middle-income countries with lack of expertise and funding to establish a T&T system. Technical assistance, independent from the tobacco industry influence, for low-income countries will be necessary for a successful implementation of the Protocol at global level.

## Conclusion

The Protocol to Eliminate Illicit trade in Tobacco Products, is an international treaty with the objective of eliminating all forms of illicit trade in tobacco products through a package of measures to be taken by countries acting in cooperation with each other. By March 2021, there were 62 parties to the Protocol.

The opportunities presented by this new international treaty are evident. The elimination of the global illicit tobacco trade would lead to more revenue and less tobacco sales. Elimination of illicit tobacco trade can have a positive impact on public finances and should be part of the COVID-19 recovery plan in each party.

But the challenges cannot be ignored either. Less economic growth, more financial constraints, travel restrictions and the subsequent annulment of all physical meetings as a result of COVID-19 made the implementation of the Protocol more difficult.

While some countries, such as India, are trying to move forward with the implementation of the Protocol, others have noted increased levels of illicit trade during the pandemic.

The sudden ban of tobacco products to tackle COVID-19 in South Africa was not a brilliant move. South Africa should have first controlled its illegal cigarette market by implementing a T&T system and ratifying the ITP. Tackling the illicit tobacco trade will not only improve public health, but also safeguard tax revenues so desperately needed for the recovery efforts. This holds not only for South Africa, but for each WHO FCTC party.

## References

[1] World Health Organization (2013). Protocol to Eliminate Illicit Trade in Tobacco Products.

[2] (2013). Protocol to Eliminate Illicit Trade in Tobacco Products.

[3] Joossens L, Merriman D, Ross H, Raw M (2010). The impact of eliminating the global illicit cigarette trade on health and revenue. Addiction.

[4] Goodchild M, Paul J, Iglesias R, Bouw A, Perucic AM (2020). Potential impact of eliminating illicit trade in cigarettes: a demand-side perspective. Tob Control.

[5] Research Unit on the Economics of Excisable Products.

[6] Vellios N, van Walbeek C, Ross H (2020). Illicit cigarette trade in South Africa: 2002–2017. Tob Control.

[7] Filby S, van der Zee K, van Walbeek C (2021). The temporary ban on tobacco sales in South Africa: lessons for endgame strategies. Tob Control.

[8] National Income Dynamics Study – Coronavirus Rapid Mobile Survey.

[9] McLaggan M (2020). When the Smoke Clears: The ban on tobacco products in South Africa during COVID-19.

[10] World Bank Group Confronting Illicit Tobacco Trade: a Global Review of Country Experiences: Confronting Illicit Tobacco Trade: a Global Review of Country Experiences (English).

